# Towards a universal implementation of labor companionship: a synthesis of the policy and facility environment of eight low-and-middle income countries

**DOI:** 10.3389/frhs.2025.1550473

**Published:** 2025-07-23

**Authors:** Soha El-Halabi, Andrea Barnabas Pembe, Alexandre Dumont, Ana Pilar Betrán, Charles Kaboré, Effie Chipeta, Guillermo Carroli, Helle Mölsted Alvesson, Hussein Kidanto, Jean-Paul Dossou, Kristi Sidney Annerstedt, Lenka Beňová, Mechthild M. Gross, Peter Waiswa, Pisake Lumbiganon, Quoc Nhu Hung Mac, Meghan A. Bohren, Claudia Hanson

**Affiliations:** ^1^ Department of Global Public Health, Karolinska Institutet, Stockholm, Sweden; ^2^ Department of Obstetrics and Gynaecology, College of Medicine, Muhimbili University of Health and Allied Sciences, Dar Es Salaam, Tanzania; ^3^ Research Institute for Sustainable Development, Université Paris Cité, IRD, Inserm, Ceped, Paris, France; ^4^ UNDP/UNFPA/UNICEF/World Bank Special Program of Research, Development and Research Training in Human Reproduction (HRP), Department of Sexual and Reproductive Health and Research, World Health Organization, Geneva, Switzerland; ^5^ Institut de Recherche en Sciences de la Santé (IRSS), Ouagadougou, Burkina Faso; ^6^ College of Medicine, The Centre for Reproductive Health, University of Malawi, Blantyre, Malawi; ^7^ Centro Rosarino de Estudios Perinatales, Rosario, Argentina; ^8^ Department of Obstetrics and Gyneacology, Aga Khan University, Medical College, Dar es Salaam, Tanzania; ^9^ Centre de Recherche en Reproduction Humaine et en Démographie (CERRHUD), Cotonou, Benin; ^10^ Department of Public Health, Institute of Tropical Medicine, Antwerp, Belgium; ^11^ Midwifery Research and Education Unit, Hannover Medical School, Hannover, Germany; ^12^ Department of Health Policy Planning and Management, Centre of Excellence for Maternal Newborn and Child Health, School of Public Health, Makerere University, Kampala, Uganda; ^13^ Department of Obstetrics and Gynaecology, Faculty of Medicine, Khon Kaen University, Khon Kaen, Thailand; ^14^ Pham Ngoc Thach University, Ho Chi Minh City, Vietnam; ^15^ Gender and Women’s Health, Nossal Institute for Global Health, School of Population and Global Health, University of Melbourne, Melbourne, VIC, Australia; ^16^ Department of Disease Control, London School of Hygiene & Tropical Medicine, London, United Kingdom

**Keywords:** LMICs, labor companionship, implementation research, health policy, maternal health, quality of care, childbirth, intrapartum care

## Abstract

**Background:**

Labor companionship, the presence of a woman's person of choice during childbirth, has benefits to both woman and baby and is recommended by the World Health Organization since 2012. However, implementation remains sub-optimal, especially in low-and-middle-income countries (LMICs). This study aimed to understand the maturity of labor companionship implementation in eight low-and-middle income countries with focus on the policy and facility environment.

**Methods:**

This was a multi-country study nested in two hospital-based implementation research studies: Action Leveraging Evidence to Reduce perinatal mortality and morbidity in Sub-Saharan Africa (ALERT) study and the QUALIty DECision-making by women and providers for appropriate use of caesarean section (QUALI-DEC) study. We included 48 hospitals from eight countries: Argentina, Burkina Faso, Thailand and Viet Nam (QUALI-DEC) and four from each of Benin, Malawi, Tanzania and Uganda (ALERT). We used data from (i) a document review, including national policy documents and (ii) health facility readiness assessment, including physical layouts of maternity wards, all collected between December 2019 and April 2021. Our analysis included two steps, (1) a structured data abstraction with coding to pre-defined categories to analyse the national polices and available resources on a facility level which informed the (2) categorization of implementation maturity in three implementation phases modelled by the framework by Bergh et al. and the logic model developed by Bohren et al.

**Results:**

Three of the eight countries lacked any national-level companionship policies, four had some mentioning and only one had detailed guidance on roles of labor companions and implementation guidelines. The physical outlines of maternity wards varied greatly, and lack of space was one of the main implementation barriers to all countries except Argentina. We classified Benin, Thailand and Viet Nam in the pre-implementation phase because of missing guidelines and limited implementation; Burkina Faso, Malawi, Uganda and Tanzania in the early implementation phase; and Argentina in the institutionalization phase where policies and facility resources were conducive.

**Conclusion:**

Successful implementation was supported by concrete and contextualized implementation guidance. To move to high implementation levels, supporting policies, guidelines and structural changes in the maternity wards are needed.

## Introduction

Continuous support for a woman throughout childbirth by a partner, family, community member or friend, referred to as “labor companionship”, is an effective evidence-based intervention which can contribute to respectful, dignified and safe childbirth (1). A labor companion is often a person who has a trusting relationship with and is chosen by the pregnant woman. There is strong evidence that labor companionship improves the health and well-being of women and babies, including reduced caesarean section rates, lower use of intrapartum analgesia, shortened duration of labor, better five-minute APGAR scores and improved overall experience of childbirth (2).

Labor companionship is a critical component of the World Health Organization (WHO) framework on the quality of maternal and newborn care, with equal importance given to women's and newborns' experiences of care and the provision of safe and respectful care (3). Autonomy and choice for all women throughout childbirth, including their wishes to have a labor companion or not, are recommended in several WHO recommendations and standards (1, 3–6) and other frameworks such as the framework for maternal and newborn care by Renfrew et al. (7).

Despite the evidence of benefits, the implementation of labor companionship is sub-optimal across settings, especially in low- and middle-income countries (LMICs) (8). A wide variation in coverage of labor companionship was reported in a recent scoping review, with almost one-third of included studies reporting less than 40% coverage and another one-third between 40%–80% (8). In some contexts, implementation is facilitated through the presence of legal frameworks providing all women the choice to be accompanied by a labor companion, while in others labor companions are allowed in specific stages of birth or not at all (9–11).

Several factors hindering implementation of labor companionship were highlighted in a Cochrane qualitative evidence synthesis, including (i) constrained physical space, (ii) limited privacy, and (iii) lack of training for healthcare providers, women, and potential companions (12). The notion of labor companionship as “less important” compared to other areas of quality care among key implementors and policy makers was also identified as a barrier (12). This was further highlighted during COVID-19 when labor companionship was not offered or actively prohibited to women in various settings despite WHO's recommendation to do so (13–15), reflecting a relative de-prioritisation of this essential evidence-based practice and its benefits. This underscores the importance of an enabling policy environment and the integration of companions in routine childbirth care (12). In summary, this evidence points to the links between the policy context and facility environment to appropriately implement labor companionship.

Implementation is portrayed as a process that includes multiple phases and processes (16). For example the model by Bergh et al.,—known in the field of maternal health—conceptualizes implementation as three phases: pre-implementation, implementation and institutionalization (17, 18). To date, research on implementation of labor companionship in LMICs has typically focused on the acceptability of this practice with emphasis on women's and healthcare providers' perspectives (19–21). Little is known on the progression of labor companionship implementation in various settings and underlying conditions of variability (9, 22). With a view to inform policy and practice of companionship during labor and childbirth in LMICs, our study aimed to understand the maturity of labor companionship implementation in eight low-and-middle income countries with focus on the policy and facility environment.

## Materials and methods

This was a multi-country, hospital-based study nested in two implementation research studies: the Action Leveraging Evidence to Reduce perinatal mortality and morbidity in Sub-Saharan Africa (ALERT) study (23) and the QUALIty DECision-making by women and providers for appropriate use of caesarean section (QUALI-DEC) study (24). Both studies ran from January 2020 until December 2024.

The ALERT study aimed to reduce facility-based perinatal morbidity and mortality and is implemented in sixteen hospitals in total, four in each of the four participating countries: Benin, Malawi, Tanzania, and Uganda. ALERT's components included (i) co-design of intervention with end-users; (ii) in-service midwifery competency-based training; (iii) quality improvement in the maternity wards and (iv) leadership mentoring in maternity units (23). Labor companionship was a key theme under the co-design and competency-based training components, with the aim of designing relevant interventions with women, their companions and healthcare providers.

The QUALI-DEC multifaceted study aimed to optimize decision-making of mode of birth in thirty-two hospitals in total, eight in each of the four participating countries: Argentina, Thailand, Viet Nam and Burkina Faso. QUALI-DEC includes (i) opinion leaders who promote evidence-based clinical guidelines for labor and birth management; (ii) audit and feedback of caesarean sections to help healthcare providers identify areas for improvement; (iii) a Decision Analysis Tool that guides women to make an informed decision on mode of birth; and (iv) implementation of labor companionship as per the WHO recommendations (24). As part of the QUALI-DEC study, a labor companionship model has been codeveloped in each of the hospitals.

### Setting

We included a total of 48 hospitals in eight countries. The hospitals were a mix of public, private-for-profit, and faith-based and offered routine and emergency peripartum and perinatal care. The number of births per month per hospital varied across countries from <100 births in Argentina to around 1,000 births in Viet Nam. Doctors were the main healthcare providers of routine childbirth care in Argentina and Thailand, as opposed to the other countries where midwives and nurse-midwives were the main healthcare providers of childbirth care. All hospitals reported facing challenges with availability of staff in relation to number of births, except in Argentina. Hospitals in Benin, Malawi, Uganda and Tanzania did not have medical doctors during night shifts; instead, doctors were on call in case of difficult labor and birth (Table 1).

**Table 1:** Characteristics of eight countries and 48 hospitals included in the study.

		Argentina	Benin	Burkina Faso	Malawi	Uganda	Tanzania^g^	Thailand	Viet Nam
Country level indicators									
Country's income group^a^ (2022)		Upper-middle	Lower-middle	Low	Low	Low	Lower-middle	Upper-middle	Lower-middle
% of births in hospitals^b^		100	35.4	80.0	42.2	47.8	47.8	99.0	94.0
% births by CS^c^		29.1 (2011)	5.3 (2014)	3.7 (2015)	6.1 (2016)	5.3 (2011)	5.9 (2016)	32.7 (2016)	27.5 (2013)
Maternal mortality ratio^d^ (2020)		45 (38–53)	523 (397–768)	264 (169–394)	381 (269–543)	284 (191–471)	238 (174–381)	29 (24–34)	124 (81–190)
Hospital beds^e^		49.92 (2017)	5 (2010)	4 (2010)	13 (2011)	5 (2010)	7 (2010)	21 (2005)	31.8 (2013)
Hospital level indicators (*n* = 48)^f^									
Main provider of routine childbirth care		Medical doctor	Midwife	Midwife	Nurse-midwife	Midwife	Nurse-midwife	Medical doctor	Midwife
Number of hospitals		8	4	8	4	4	4	8	8
Sector	*Public*	8	3	8	3	3	3	8	6
	*Private*	0	0	0	0	0	0	0	2
	*Faith-based*	0	1	0	1	1	1	0	0
Level	*Primary*	0	0	2	0	0	0	0	2
	*Secondary*	4	3	4	4	4	4	2	4
	*Tertiary*	4	1	2	0	0	0	6	2
Monthly number of births^f^ -median (IQR)		91 (61, 144)	181 (163, 259)	305 (205, 426)	303 (236, 401)	346 (201, 475)	156 (130, 200)	376 (311, 409)	853 (489, 1,247)
Midwifery/nursing staff^f^ -median (IQR)									
Day shift		7 (4, 11)	N/A	3 (3, 3.87)	3.5 (3, 5.5)	2.5 (2, 3)	5.5 (5, 6.75)	10.5 (8, 11)	9 (3.75, 14.25)
Night shift		4 (2, 5)	N//A	3 (3, 3.87)	3 (2.25, 3.75)	2 (2, 2.75)	2 (2, 2.75)	8 (6, 8.75)	9 (3.75, 11.5)
Medically/ Surgically trained staff^f^ -median (IQR)									
Day shift		8 (7.5, 15.5)	N/A	1 (1, 3.75)	3 (2.25, 5.25)	On call	2 (2, 2.75)	7 (5.5, 10.25)	3 (1, 6.5)
Night shift		8 (0, 8.5)	On call	1 (1, 3.75)	On call	On call	On call	6 (4, 8.5)	3 (1, 6.5)

^a^ World Bank's classifications (49).

^b^ Study data sources (23, 24).

^c^ Based on data from the Global Health Observatory (50).

^d^ Expressed per 100,000 live births (51) with uncertainty interval.

^e^ Measured per 10,000 population and based on data from the Global Health Observatory (52).

^f^ Based on study data from health facility assessments, IQR is based on sample of hospitals in the given country.

^g^ In Tanzania there are three shifts- morning and day shifts were merged when calculating number of available staff; CS: caesarean section.

### Data sources

We used data included in the formative research of the QUALI-DEC (25) and ALERT studies. Data collection was done by the country implementation teams and trained data collectors and included the following sources (i) a document review covering relevant national policies, protocols, and guidelines about maternal and newborn health; and (ii) a formal health facility readiness assessment, including data on number of available healthcare providers, number of rooms, beds and toilets per maternity ward. The health facility readiness assessments also included a section on understanding companionship practices in the hospitals, with data generated on stages (labor or birth) where companions were allowed to be present, who was allowed to be a companion, where they stayed (with woman or outside maternity ward) and a description of their roles. These questions required both observations of the labour ward and a discussion with staff members. Data were collected between March 2019 and April 2021; more details are provided under Supplementary File 1.

An additional search to identify policy documents in Benin, Malawi, Tanzania and Uganda was done as part of our analysis. National repositories of Benin, Malawi, Tanzania and Uganda (i.e., websites of Ministries of Health) were searched to identify, map and review national level policies pertaining to labor companionship (for ALERT countries). Additionally, we iteratively interviewed ALERT country research teams, including senior obstetricians and midwives, for further information regarding the national guidelines/policies and maternity ward layout in each of the ALERT hospitals.

### Analysis

First, we analysed the content of available national policy documents in the eight countries in ALERT and QUALI-DEC. We reviewed relevant national policies, manuals and training materials which included labor companionship. We performed a directed content analysis with coding to pre-defined categories including: (1) benefits of having a labor companion; (2) roles of a companion; and (3) integration of labor companions in routine childbirth care. Information that corresponds to these pre-defined categories were highlighted and included in a spreadsheet (Supplementary File 2). Summary findings including similarities and differences in the content of these policies were drafted. These categories were agreed on by co-authors and the data abstraction was performed by the first author. Identified policies and abstracted data were cross-checked with consortium members who were senior clinical experts within the two studies and further policies were suggested for review at the country level. Cross-checking of findings was done through the presentation of the preliminary analysis through (i) online meetings with representatives from each country team under the two studies and (ii) discussions with all authors of this paper on the representativeness of these results.

Additionally, we described the implementation of companionship practices on a hospital level. We prepared a separate extraction sheet to document the number of rooms per maternity ward, sources of privacy (i.e., types of separators), presence of private and shared toilets and bathrooms and stages (i.e., labor and birth) where companions were allowed to be present. The sheet was populated based on our data from the readiness assessments and insights from country teams when data were not available.

**Figure 1:**
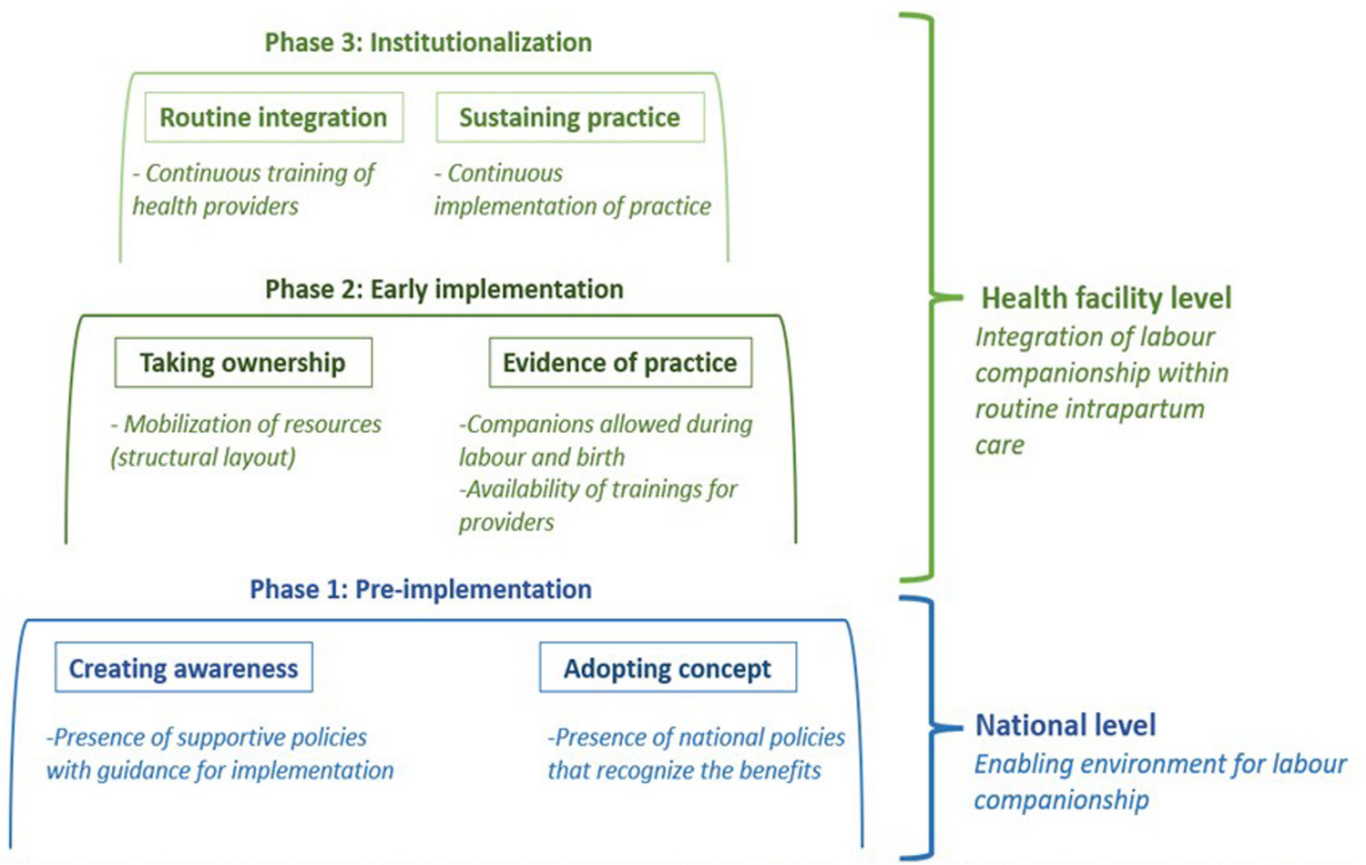
Illustration of adapted model integrating the stages of implementation by Bergh et al. and the logic model by Bohren et al. In this figure, we colour coded national level factors in blue and those pertaining to healthcare facility level in green. We used two degradations of green colour to distinguish between the two implementation phases: early implementation and institutionalization. We included in rectangles the constructs as highlighted in the model by Bergh et al. and in italic the factors as presented in the logic model by Bohren et al.

In a second step, using the findings from the steps above we categorised the countries following the work by Bergh and colleagues (17). We classified countries into three implementation phases: (1) *pre-implementation—*documents were not available on a national level and labor companionship was only sporadically offered in labor rooms; (2) *early implementation—*policies were available on a national level but their implementation varied (i.e., some hospitals allowed a companion to be present in labor or delivery room and some did not), and (3) *institutionalization*—policies on a national level existed and women were allowed to have companions in labor and delivery rooms. To further understand the implementation phase of labor companionship, we focused on factors of successful implementation highlighted by the model by Bohren et al. (12). The model highlights essential components for a successful implementation of labor companionship, including enactment of formal policies, physical spaces of maternity wards where privacy is maintained, and proper integration of companions in routine care (Figure 1).

We sketched the physical layout in maternity wards by implementation level based on pictures of the maternity wards taken as part of the facility assessments. The sketches were initially developed by the first author (SEH) per country and presented to the country teams. After the initial approval of country based sketches, they were aggregated per implementation phase to describe measures of privacy, hygiene, labor companionship and areas where food and drinks were allowed. The aggregated sketches were cross-checked with country team members in both studies to ensure that they were representative of the settings. Subsequent refinement was done with feedback from authors, particularly the number of icons referring to women was changed to match the average number of women in a room.

## Results

### Synthesis of available national policies

A total of 11 country-level documents were identified and analysed; all identified documents with references are available as Supplementary File 3. All identified documents were published between 2015 and 2020, except for Argentina, where the law was passed in 2004. We could not identify any national policy documents with reference to labor companionship in Benin, Thailand and Viet Nam. However, in Benin, labor companionship was mentioned in educational material of prenatal classes for women.

Malawi was the country with the highest number (*n* = 4) of documents mentioning labor companionship, including a national strategy and guidelines (26, 27). In Tanzania, we identified one national guideline on *Gender and Respectful Care Mainstreaming and Integration across Reproductive maternal, newborn, child and adolescent health services* (28). The guideline included good practice code for birth companionship programme that was piloted at a hospital level. Burkina Faso had a protocol on reproductive health including implementation of labor companionship. We also identified guidelines developed for maternal and newborn care during the COVID-19 pandemic in Malawi and Uganda, both of which encouraged healthcare providers to allow women to have a labor companion of their choice through labor and birth (27, 29). The document from Malawi, specified measures for labor companions including (i) not roaming around the maternity ward and (ii) wearing a face mask (27).

#### Acknowledging the benefits of the practice

Labor companionship was recognized as an important practice for the safety and well-being of women during childbirth in two documents identified from Malawi, Burkina Faso and Argentina. Argentina was the only country with a law on humanized birth, which included the right to labor companionship. The presence of a labor companionship was considered to be a way to establish a “*bond of containment and continuous support*” leading to better experiences and outcomes for women in labor such as shortening duration of labor and requiring less medication and analgesia (30).

Identified documents clearly emphasized women's choice and autonomy for every woman. Country documents defined a companion as an “…*accompanying person chosen by the pregnant woman…*” [Argentina, (31)]; “*the companion of her choice*” (32); and “*person of choice*” [Uganda, (33)]. In Tanzania a companion was restricted to “*a female, non-medical person…*” (28).

#### Potential roles of labour companions

Only country documents from Burkina Faso, Tanzania and Argentina provided guidance on the nature of labor companions' roles.

“The birth companion's primary role is to provide continuous emotional support to a woman throughout labor and delivery. Birth companions also provide practical, physical, and informational support as well as serve as an advocate for the woman throughout pregnancy, labor, delivery and the postpartum period.” (28).

In Burkina Faso, labor companions were expected to act as a link between the family and healthcare providers. The country document provided a list of practical tasks that labor companions could do to support women such as, bringing food and drinks to the woman, purchasing medication, and bringing lab results (34).

The role of labor companions was explained more concretely in the national document from Argentina which stated that the companions play a role in emotionally supporting the woman.

“…try to improve the mother's control verbally or with affectionate demonstrations: holding her hands, massaging her back, accompanying her wandering…” [Translated from (30)]

#### Integration of labour companions in routine childbirth care

Most documents, except those from Argentina, did not explicitly describe ways of integrating labor companions in routine childbirth care. In Burkina Faso and Uganda, women developed a childbirth plan with their healthcare providers, including identification of their labor companion (29, 34).

The documents from Argentina included clearer and specific guidelines around the inclusion of the family/companion of choice in the delivery room, joint hospitalization, long visiting hours, and residences for mothers were explained:

“The health care team and the health care institution shall provide the woman and her companion with information about the physiological and vital processes involved in pregnancy, labor, birth as well as the role of the health team.” [Translated from (31), Argentina]

### Physical environment of maternity wards

There were differences in the policies and structure of maternity wards across the facilities in the eight countries. Table 2 presents a summary of facility level resources and companionship practices across the health facilities in the eight countries based on data from the facility health assessments in the two projects.

**Table 2:** Facility level resources and companionship practices across the health facilities in the eight countries.

	Argentina	Benin	Burkina Faso	Malawi	Tanzania	Thailand	Uganda	Viet Nam
Companions allowed in								
Labor	In 5/8 hospitals	In 1/4 hospital	No	In all hospitals	In 1 hospital	In 2/8 hospitals	In all hospitals	In 7/8 hospitals
Birth	In 5/8 hospitals	No	No	No	In 1 hospital	No	In 2/4 hospitals	Private room only
Labor rooms								
Number^a^	1 (1–3)	1	1	1	1	14 (11–20)	1	2 (1–10)
Separators	If sharing (3/8 hospitals)	No	No	Yes	No	In all hospitals	No	In 1 hospital
Types of separators	Mobile screens, partitions, curtains	N/A	N/A	Curtains	N/A	Curtains	N/A	Curtains
Shared toilet/washroom	No, unless woman is sharing room	Yes	With companion, outside the hospitals	Yes	Yes	Yes, unless woman had private room	Yes	Yes, unless woman had private room
Delivery rooms								
Number^a^	2 (1–4)	1	1 (1–2)	1	1 (1–4)	4 (3–20)	1	2 (1–7)
Separators	In all hospitals	In all hospitals	In 5/8 hospitals	In all hospitals	In all hospitals	In 4/8 hospitals	In 2/4 hospitals	In 4/8 hospitals
Type of separators	Cubicles divided by walls, fixed partitions, and closed doors	Mobile screens and curtain	Cubicle, mobile screens, partition, curtain, concrete walls with no curtains	Mobile screens	Cubicles, mobile screens and curtains	Curtain	Curtain and mobile screens	Curtain
Shared toilet/washroom	No, unless woman is sharing room	Yes	Yes, with companions, outside the hospitals	Yes	Yes	Yes, unless woman had private room	Yes	Yes, unless woman had private room
Private rooms available	Yes, no extra fee	No	No	No	No	In 3 hospitals for extra fee	In all hospitals for extra fee	In 3 hospitals for extra fee
Companions allowed in postpartum	Yes	Yes	Yes	Yes	In case of complication or CS	Yes	Yes	Yes
Designated areas for companions	Stay with women	Outside labour ward	Outside labour ward	Outside labour ward	Outside labour ward	Yes	Outside labour ward	Yes

^a^ Expressed in median (IQR), based on data from the health facility assessments.

In the absence of guiding policy documents in Benin, Thailand and Viet Nam, the implementation of labour companionship was rather sporadic. Hospitals in Benin, Thailand and Viet Nam could not systematically accommodate companions in labor or in delivery rooms due to overcrowding of maternity wards which limited privacy. Hospitals mostly used separators in the delivery room but not necessarily in labor rooms. In Thailand and Viet Nam, companions were allowed in labor rooms, in which women were staying during early labor, only when the rooms were not crowded. The availability of private rooms for an extra fee was also common in Thailand and Viet Nam, which then allowed the presence of companions with the women. In Benin, on the contrary, people accompanying birthing women to the hospital waited outside the maternity wards and were only allowed to attend to women in postpartum rooms. In one of the hospitals, there was a designated place for people accompanying birthing women, facing the maternity ward. However, based on insights from the implementing team, this space was rarely used as people accompanying birthing women preferred to wait at the door of the maternity ward. A midwife usually called for them to buy medication or other necessary items for childbirth. Only female labor companions were allowed, except for some hospitals in Thailand.

While policy documents in Uganda, Burkina Faso, Malawi and Tanzania emphasized on the practice being universally offered for every woman with the opportunity to choose a suitable companion, the implementation on a facility level was not aligned. Companionship was not offered to every woman, due to overcrowding, which was a particular challenge in the maternity wards in Burkina Faso where women often gave birth in the corridors due to lack of space. The practice was restricted to females only and in the case of Burkina Faso women had limited control over whom they chose, as it was rather the family who chose, mostly a mother- or sister-in-law or a sister (11). Labor companions were not allowed in delivery rooms; instead, they waited outside the hospital premises. Even though private rooms were offered for an extra fee in Uganda, a companion could still not be present during birth Instead, these private rooms were only used during labor and postpartum periods and women were moved to public delivery rooms for childbirth. Women in private rooms received the same level and type of services but were offered more privacy. Private rooms did not include extra beds or chairs for labor companions.

There was a difference in the implementation level of the practice in Argentina. Labor companionship was recognized as an evidence-based practice and an important aspect of humanized childbirth care. Women had privacy during labor and birth and could be accompanied by their person of choice. The role of labor companions shifted from practical tasks towards emotionally supporting the woman through the different stages of birth. Women could choose their companion and were not limited to having a woman, except in one hospital. Where women had to share a room, separators in the labor and delivery rooms were available.

### Implementation phases of labor companionship

Based on these characteristics, we classified the eight countries into three different categories, pre-implementation; early implementation; and institutionalization.

We classified Benin, Thailand, and Viet Nam in *pre-implementation phase* since labor companionship was not a priority on the national political agenda and the implementation of the practice was solely left to the hospitals. Implementation remained inconsistent (with allowed labor companions being mainly women) and depended on each hospital's resources with minimal measures to ensure privacy (Table 3).

**Table 3:** Distribution of countries according to the three phases of implementation.

Phase	Country	Key characteristics
1-pre-implementation	Benin, Thailand, and Viet Nam	• No documents identified on the national level • If allowed labor companions in hospitals were women and allowed to spend (some) time with the laboring woman, not necessarily during birth. • Crowded hospital maternity wards, privacy not maintained • Labor companions were allowed in private rooms for an extra fee • No training on hospital level for healthcare providers on integration of companions • No clear roles and responsibilities for companions
2-early implementation	Burkina Faso, Malawi, Uganda, and Tanzania	• Identified policy documents mention the importance of labor companionship • Labor companions in hospitals are women and allowed to spend time with the laboring woman but not consistently throughout birth • Crowded hospital maternity wards, privacy not maintained • Labor companions are allowed in private rooms for an extra fee • Inconsistent training/orientation of healthcare providers at hospital level on integration of companions • No clear roles and responsibilities for companions (except for Burkina Faso)
3-institutionalization	Argentina	• Labor companionship is recognized as every woman's right and protected by law • Women choose their labor companions who are allowed in delivery rooms (most of the time) • Privacy maintained throughout labor and birth • Healthcare providers are trained on integration of companions • Roles and responsibilities for companions are set and communicated to the companions

Four countries were classified in the *early implementation* phase—Burkina Faso, Tanzania, Malawi and Uganda. In this phase, the benefits of labor companionship were reflected in national- and local-level policies. However, implementation of labor companionship in these hospitals was somewhat limited, mainly due to limited physical space considering the volume of births, and therefore challenges in maintaining privacy. Labor companions were mostly women (hospitals' rules) and accompanied women during labor, and occasionally during vaginal birth.

One country (Argentina) was classified in the *institutionalization phase,* where a civil law was passed recognizing labor companionship as a right with emphasis on autonomy. Hospitals offered each woman the opportunity to be accompanied by a person of choice. The emphasis of national policy was for women to benefit from continuous emotional support. In most hospitals, women could choose who their companion was and were not limited to having women as labor companions (Table 3).

### Maternity ward sketches by implementation phase

**Figure 2:**
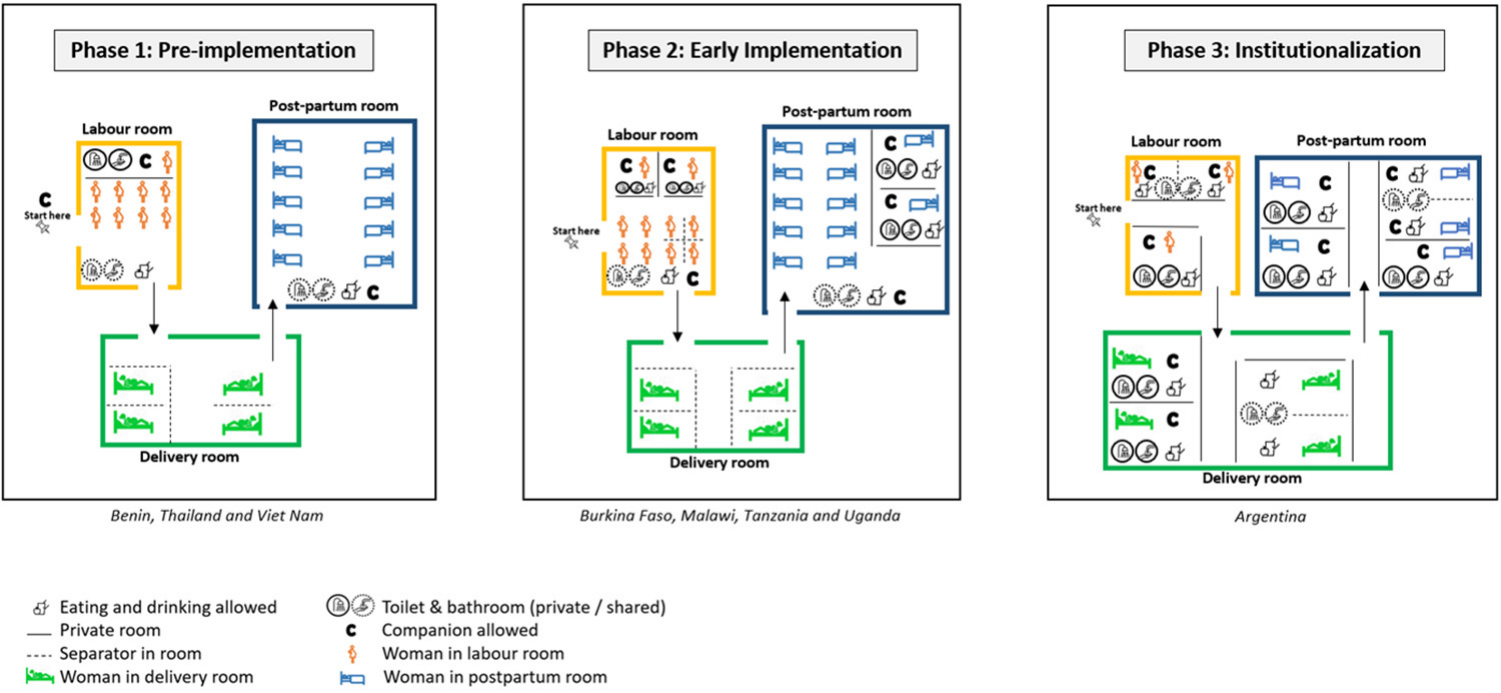
Sketches depicting the characteristics of maternity wards by implementation phases.

Comparing countries categorised as early implementation (phase two) to those categorised as phase one (pre implementation), there were notable differences in privacy measures (i.e., private rooms and separators within the shared room) introduced (Figure 2). The number of women within a shared room also decreased from phase one to phases two and three. This translated to more labor companions being able to support the women especially in labor rooms. In phase three, women had more privacy across rooms, which meant that labor companionship could be practiced across different stages of birth. Food and drinks were generally restricted in delivery rooms in phases one and two, but this is not the case for phase three where women had more space. In three countries including Benin (phase one, pre-implementation), Burkina Faso (phase two, implementation) and Argentina (phase three, institutionalization), health facilities offered prenatal classes for women to attend during pregnancy. These classes covered topics around the roles of labor companions and people accompanying birthing women. Only hospitals in Argentina provided trainings to healthcare providers on labor companionship at baseline.

## Discussion

We compared the policy and facility environments in eight countries to better understand the interconnectedness between the policy and facility environment supporting the implementation of evidence-based practices such as labor companionship. While some type of labor companionship has long been practiced in community and healthcare settings, our analysis indicated that a systematic and rigorous approach to companionship is recent on the health policy agenda of the LMICs.

Countries classified in the early implementation phase presented a disconnect between the national policies and their translation to the hospital level. Most of the identified national documents emphasized every women's right to choose her labor companion, yet in practice labor companions were not allowed to be present during childbirth at all times. Similar findings from other LMICs have been reported, highlighting restrictive hospital policies as a challenge towards the implementation of labor companionship (35, 36). It may also be a consequence to the strict top-down approach of policy development in these settings, which is not the case in Argentina for example, where more involvement of the civil society exists (37). While a hospital level guideline on labor companionship is necessary to promote the practice, it is not sufficient on its own. The enactment of national policies on hospital level remains a challenge. Policies need to be adapted to fit the context and accompanied by sufficient resources, training and an organizational readiness for change (38, 39). Further, it is important that women's voices and preferences are considered, especially since few of the policies refer to women's autonomy.

Our analysis underscores the importance of physical spaces for the implementation of labor companionship. In many settings, maternity wards were overcrowded shared spaces with minimal efforts and abilities to maintain privacy. This often translated to men not being allowed in maternity wards to maintain the privacy of other women. Moreover, overcrowding also led to intermittent rather than continuous labor companionship throughout the continuum of labor and birth for all women. Maintaining privacy is a common challenge in many LMICs (10, 40–44), making maternity wards not responsive spaces to women's needs. Insufficient investment in the physical layout of maternity wards- independent of the country's economic wealth- is a key underlying reason for this and may be related to the perception among decision makers and healthcare providers that labor companionship is not an important intervention in maternal health but rather a luxurious opportunity for those who can afford it. This notion was further reflected in some hospitals where the continuous presence of a labor companion was made possible for an extra out-of-pocket fee for a private room.

Identified policy documents did not include clear guidelines on the integration of labor companions during labor and birth, except for Argentina. Integrating companions in labor and birth care might allow labor companions to effectively support the women and enhance their communication with the healthcare team (12). This integration can face resistance from healthcare providers, including the limited time and resources to train healthcare providers in understaffed facilities, healthcare providers' fear of infection in labor wards and risk of litigation (10, 12). Moreover, in some cases, women have limited control over their choice of companions which may create extra burden. In many cases, labor companions are pre-assigned (e.g., an in-law) and women do not get the opportunity to choose a person of their choice (11). Thus, it becomes more important to facilitate the integration of labor companions that the women choose.

### Implications for policy and practice

The integration of labor companionship as a practice in routine childbirth care including the systematic tailoring of the content to a hospital level would be beneficial for countries in phase one and two. This can be reflected in the presence of clear roles for companions and available trainings for healthcare workers and women and their companions on their roles during childbirth. While considering the evidence supporting labor companionship, the design of the policies and the implementation processes should engage and remain centred on the end-users (women, new-born, companions, and healthcare providers). This requires that system thinking principles underline the design and implementation, meaning that labor companionship should be viewed as a component of the whole maternity care package and not as a stand-alone intervention. Frequent context specific adaptations should be feasible to ensure the whole process remains centred on the users in a sustainable way.

Hospital level policies that promote companions of choice, of any gender to be continuously present in maternity wards, need to be in place. As part of the two studies, particularly through the two components on opinion leader engagement (in QUALI-DEC) (24) and co-design (in ALERT) (23), there is an opportunity for the hospital teams, women and their companions to propose strategies that address implementation barriers and integrate labor companionship (10, 11). As part of the intervention implementation, Information, Education and Communication material have been developed within the two studies after the baseline assessment. These are important in promoting, informing, and educating healthcare providers, women and their companions in the relevant context.

The restructuring of the physical layout of maternity wards is essential. Kabakian et al., highlight simple interventions such as adding curtains and chairs for companions (45) which is likely needed for hospitals in pre-implementation and early implementation phases. Maternity wards in early implementation wards require privacy measures including curtains or other types of separators while maintaining possibility of easy view to cater for the low ratio of healthcare providers and women in labor. The literature highlights the importance of investing in hospital birthing rooms that support women's psychological needs while also meeting care providers' requirements in their work environments (46, 47).

In our study we integrated the two models by Bergh et al. and Bohren et al., to accommodate for the use of both national and facility-based data. We call for the integration of top-down and bottom-up approaches (engaging midwives, women and their companions) to contextualize and sustain this evidence-based practice. Moreover, we recommend that process indicators are included to comprehensively capture the implementation of labor companionship on local levels e.g., data on proportion of women who had a labor companion during labor, birth and postpartum as suggested by Bohren et al. (8).

### Strengths and limitations

The categorization of the countries across phases allowed for a better understanding of the implementation processes and highlighted the interconnectedness of the policy and facility environments. Our use of a subject-specific framework with components and constructs that are more relevant to labor companionship in comparison to other available generic frameworks enhanced our understanding of implementation maturity in the eight countries. We acknowledge that our results are derived from eight countries only, and a limited number of facilities per country may not represent the national landscape. However, our study included a range of facility types (private, public, faith-based) and characteristics. Thus, we believe that the results present a sufficiently good idea of typical maternity ward layouts. This was also confirmed by the research teams in the countries who have long working experience in different geographical areas and healthcare facilities within the study countries.

We presented a rather “linear” direction of implementation which is a simplified reflection of implementation practices. We did not use the scoring component as provided by Bergh et al. because (i) this model was initially used to assess the implementation of kangaroo mother care at the facility-level, (ii) progress markers used may not have been suitable to understand labor companionship practices, (iii) and we included a mix of national and facility indicators. We acknowledge that in real life scenarios, implementation is multidimensional and could regress due to many factors such as funding, health financing policies, health system disruptions, architecture of maternity wards and the organizational culture of hospitals. Further, due to the data available at the time of analysis we did not include macro-level factors such as, competing policy agendas or availability of funding.

We also acknowledge that the resources and functionality of health systems across the included countries varies which may result in variability of implementation across facilities within a country. While the availability of resources is necessary for the implementation of companionship, it is not sufficient, and our analysis highlights the importance of bottom-up approaches for change. For example, even in healthcare systems with relatively more resources, e.g., Thailand and Viet Nam, companionship was not implemented or introduced on a national level. Its implementation on a facility level was superficial and conditional (i.e., when rooms were not crowded, or a private room was available). This might be because companionship is perceived as a luxurious practice (i.e., women who can afford a private room) or less important compared to other clinical interventions. Labor companionship practices may also reflect power imbalances, including gender power relations within the broader contexts of health systems (48), which is beyond the scope of this study.

While the implementation of labour companionship in Argentina was not universal (3 hospitals not implementing), we believe that the practice was institutionalized given its integration into the health system's routines. We decided on this categorization given that (1) the practice was formally adopted on a national level through the law on humanized childbirth and (2) on a hospital level it was supported by relevant structures (physical structure of maternity ward), training of healthcare providers and presence of roles of companions.

Finally, some baseline data were collected during COVID-19 pandemic which may have introduced further restrictions to labor companionship on a hospital level (e.g., restricted visiting hours or number of visitors allowed). While COVID-19 restrictions may have further restricted labor companionship, implementation was sub-optimal before COVID-19.

## Conclusion

Despite the large body of evidence on the benefits of labor companionship, this intervention remains under implemented. Even in hospitals where companionship is implemented, essential features such as allowing the woman to choose her companion may not be an option. Implementing labor companionship is a continuous process requiring supportive and clear policies at both the national and the health facility levels. The contextualization of hospital level policies and reorganization of maternity wards is needed. More specifically, maternity wards need to provide space for women and their companions to maintain privacy. This study emphasizes the need to understand policy and facility-level factors to optimize implementation and contributes to informing the implementation of labor companionship practices in eight LMICs.

## Data Availability

The raw data supporting the conclusions of this article will be made available by the authors, without undue reservation.
