# Supplementary material for: Towards a universal implementation of labor companionship: a synthesis of the policy and facility environment of eight low-and-middle income countries
Source: Front Health Serv. 2025 Jul 23;5:1550473. doi: 10.3389/frhs.2025.1550473 (PMC12325283; doi:10.3389/frhs.2025.1550473)
Supplement: Supplementary file 1 [file Table1.docx]

**Duration of formative pre-intervention data collection in the eight countries**

|  | **Argentina** | **Benin** | **Burkina Faso** | **Malawi** | **Uganda** | **Tanzania** | **Thailand** | **Viet Nam** |
| --- | --- | --- | --- | --- | --- | --- | --- | --- |
| **Duration of data collection** | December 2019 | January 2021- February 2021 | February 2020-March 2020 | February 2021 – April 2021 | February 2021 | February 2021 | July 2020 – October 2020 | 2019 |
