# Supplementary figures and images for: Towards a universal implementation of labor companionship: a synthesis of the policy and facility environment of eight low-and-middle income countries
Source: Front Health Serv. 2025 Jul 23;5:1550473. doi: 10.3389/frhs.2025.1550473 (PMC12325283; doi:10.3389/frhs.2025.1550473)

**Extraction sheet developed for study analysis**


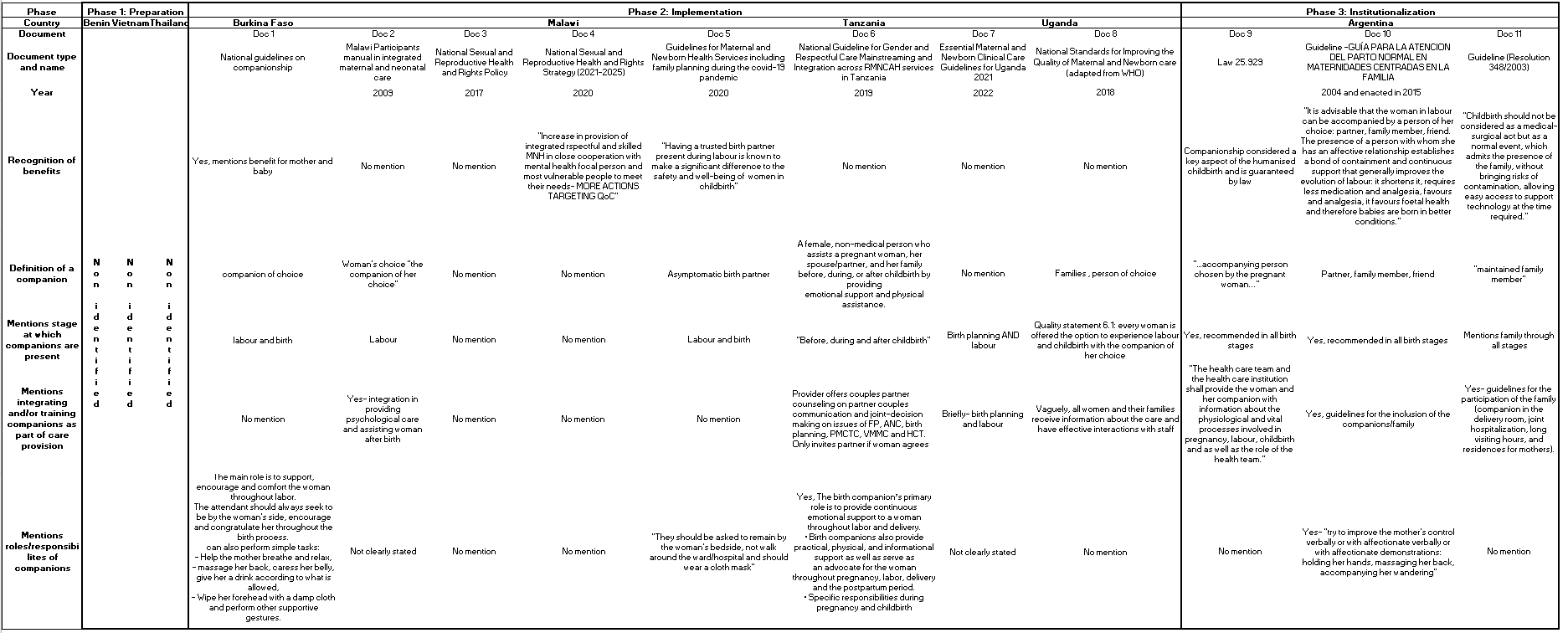

Supplement: Supplementary file 2 [file Table2.docx]
