# Supplementary material for: Towards a universal implementation of labor companionship: a synthesis of the policy and facility environment of eight low-and-middle income countries
Source: Front Health Serv. 2025 Jul 23;5:1550473. doi: 10.3389/frhs.2025.1550473 (PMC12325283; doi:10.3389/frhs.2025.1550473)
Supplement: Supplementary file 3 [file Table3.docx]

**Supplementary material on identified national documents pertaining to labour companionship per country**

| **Phase** | **Country** | **Document name** | **Year** | **Reference** |
| --- | --- | --- | --- | --- |
| 1 Pre-implementation | Benin | None identified | | |
|  | Thailand |  |  |  |
|  | Viet Nam |  |  |  |
| 2 Early Implementation | Burkina Faso | Protocole de santé de la reproduction: santé de la femme et du nouveau-né de moins de sept (7) jours | 2019 | (35) |
|  | Malawi | Malawi Participants manual in integrated maternal and neonatal care | 2009 | (33) |
|  |  | National Sexual and Reproductive Health and Rights Policy | 2017 | (51) |
|  |  | National Sexual and Reproductive Health and Rights Strategy 2021-2025 | 2021 | (28) |
|  |  | Guidelines for Maternal and Newborn Health Services including family planning during the covid-19 pandemic | 2020 | (29) |
|  | Tanzania | National Guideline for Gender and Respectful Care Mainstreaming and Integration across RMNCAH* services in Tanzania | 2019 | (30) |
|  | Uganda | National Standards for Improving the Quality of Maternal and Newborn care | 2018 | (34) |
|  |  | Essential Maternal and Newborn Clinical Care Guidelines for Uganda 2022 | 2022 | (31) |
| 3 Institutionalization | Argentina | Law 25.929 | 2004 and enacted in 2015 | (32) |
|  |  | Guia para la atencion del parto normal en maternidades centradas en la familia |  | (36) |
|  |  | Guideline (Resolution 348/2003) |  | (52) |

**: Reproductive maternal, newborn, child and adolescent health*
